# Impact of Leucine and Magnesium Stearate on the Physicochemical Properties and Aerosolization Behavior of Wet Milled Inhalable Ibuprofen Microparticles for Developing Dry Powder Inhaler Formulation

**DOI:** 10.3390/pharmaceutics15020674

**Published:** 2023-02-16

**Authors:** Shahjabeen Sharif, Saiqa Muneer, Emad L. Izake, Nazrul Islam

**Affiliations:** 1 Pharmacy Discipline, School of Clinical Sciences, Faculty of Health, Queensland University of Technology, Brisbane, QLD 4000, Australia; 2 School of Chemistry and Physics, Science and Engineering Faculty, Queensland University of Technology, Brisbane, QLD 4000, Australia; 3 Centre for Immunology and Infection Control (CIIC), Queensland University of Technology, Brisbane, QLD 4000, Australia

**Keywords:** ibuprofen wet milling homogenization, leucine, DPI formulations, aerosolization, Twin-Stage Impinger (TSI), fine particle fraction, saturated solubility, dissolution

## Abstract

This study investigated the development and characterization of leucine and magnesium stearate (MgSt) embedded wet milled inhalable ibuprofen (IBF) dry powder inhaler (DPI) formulations. IBF microparticles were prepared by a wet milling homogenization process and were characterized by SEM, FTIR, DSC, XRD and TGA. Using a Twin-Stage Impinger (TSI), the in vitro aerosolization of the formulations with and without carrier lactose was studied at a flow rate of 60± 5 L/min and the IBF was determined using a validated HPLC method. The flow properties were determined by the Carr’s Index (CI), Hausner Ratio (HR) and Angle of Repose (AR) of the milled IBF with 4–6.25% leucine and leucine containing formulations showed higher flow property than those of formulations without leucine. The fine particle fraction (FPF) of IBF from the prepared formulations was significantly (*p* = 0.000278) higher (37.1 ± 3.8%) compared to the original drug (FPF 3.7 ± 0.9%) owing to the presence of leucine, which enhanced the aerosolization of the milled IBF particles. Using quantitative phase analysis, the XPRD data revealed the crystallinity and accurate weight percentages of the milled IBF in the formulations. FTIR revealed no changes of the structural integrity of the milled IBF in presence of leucine or MgSt. The presence of 2.5% MgSt in the selected formulations produced the highest solubility (252.8 ± 0.6 µg/mL) of IBF compared to that of unmilled IBF (147.4 ± 1.6 µg/mL). The drug dissolution from all formulations containing 4–6.25% leucine showed 12.2–18.6% drug release in 2.5 min; however, 100% IBF dissolution occurred in 2 h whereas around 50% original and dry milled IBF dissolved in 2 h. The results indicated the successful preparation of inhalable IBF microparticles by the wet milling method and the developed DPI formulations with enhanced aerosolization and solubility due to the presence of leucine may be considered as future IBF formulations for inhalation.

## 1. Introduction

The pulmonary drug delivery system is one of the most preferred routes of drug administration as it delivers drugs directly into the deep lungs, avoids the first-pass metabolism, and produces therapeutic benefits at a very low dose. The DPI formulation provides deep drug deposition of drugs through the patient’s respiration force [1,2]. DPI formulations consist of inhalable sized micronized drug particles (<5 µm), which are produced by the traditional micritization method. The micronized inhalable drug particles are highly cohesive and produce a poor flow property that limits the aerosolization and efficient lung deposition of drugs. To overcome these problems, the micronized drug particles are mixed with large carriers (lactose) and dispersibility enhancers such as leucine and MgSt [1,3,4]. This study investigated the preparation of micronized particles of a model drug by a wet milling method and determined the role of leucine and magnesium stearate in aerosolization behavior for the development of an efficient DPI formulation. 

Development of microparticles with the desired inhalable size (1–5 µm) for developing an inhaled formulation is challenging. Wet milling micronization is one of the appropriate milling techniques and provides improved flowability, aerosolization and dissolution performance for the poorly water soluble drugs [5,6,7]. Numerous approaches have been reported for preparing inhalable sized particles such as spray-drying, co-milling, solvent exchange, anti-solvent precipitation, in situ micronization, and the supercritical fluid technique [8,9,10,11]. Micronization can also be achieved by common technologies such as conventional jet milling or the dry milling method, which produce large numbers of amorphous particles with high surface free energy and tend to form agglomerations due to particle–particle cohesiveness. This simply reduces the particle flowability and aerosolization behavior of the particles [11,12]. Anti-solvent precipitation is a technique to form nano/microparticles; however, particles tend to be large in size during the freeze-drying process, especially for poorly water-soluble drugs. It has been reported that the preparation of ‘nanoclusters’ (e.g., budesonide, itraconazole etc.) have provided the desired sized drug particles by different types of wet milling processes in aqueous media. 

Recently, we developed a robust wet milling method to prepare excipients containing inhalable IBF with enhanced solubility and dissolution [13]. In this study, we investigated the manufacturing of inhalable IBF microparticles (<5µm) with leucine and MgSt by our developed wet milling method, the preparation of DPI formulations, and their characterization. IBF was used as a model drug and the impact of the dispersibility enhancer leucine and MgSt on the physicochemical properties and aerosolization behavior of the prepared IBF microparticles was investigated. To ensure the stability and structural integrity of the developed IBF microparticles, the DPI formulations were stored at different temperatures and RH and analyzed by XPRD, DSC, and TGA. To determine the IBF chemical integrity, interactions with excipients, and compatibility of the drug in DPI formulations, ATR-FTIR analysis was used. 

## 2. Materials and Methods

### 2.1. Chemicals Used in This Study

USP grade Ibuprofen, Tween 80 and leucine were purchased from MEDISCA (Plattsburgh, NY, USA) and Sigma Aldrich (Castle Hill NSW, Australia). Lactose monohydrate as InhaLac^®^120 was purchased from MEGGLE group, Germany. Magnesium stearate was purchased from Professional Compounding Chemists of Australia (PCCA) Pty Ltd. (PCCA, New South Wells, Australia). Hypromellose empty capsule shells (size 3) were purchased as Vcaps^®^ Plus Capsules from LONZA Inc., New Jersey, USA. Absolute ethanol (analytical grade), acetonitrile (HPLC grade) and orthophosphoric acid were purchased form Thermo Fisher Scientific (Queensland, Australia) and, RCI Labscan Limited (South Australia Australia.), Sodium chloride, potassium chloride, potassium di-hydrogen orthophosphate and di-sodium hydrogen phosphate were obtained from MERCK Pty. Limited, Germany for preparing phosphate buffered saline (PBS, pH 7.4).

### 2.2. Methods 

#### 2.2.1. Preparation of Inhalable Sized IBF Microparticles by Wet and Dry Milling Process 

Ibuprofen original particles were micronized by a wet milling homogenization process to prepare inhalable sized IBF microparticles [13]. Then, 100 mg API was suspended in 10 mL 0.05% Tween 80 solution (*w*/*v*). The suspended mixture was sonicated for 5 min using an ultra sonicator (Branson 1510, Danbury, USA) then Tween 80 solution was added to make a final suspension up to 20 mL. Finally, the suspension was homogenized using a high shear homogenizer (Heidolph DIAX 900, Labexchange, Burladigen, Germany) at 17,000 rpm for 30 min in an iced water bath at 5–8 °C. Dry milled IBF microparticles were prepared using a mortar and pestle with the hand milling method [1,3,4]. The size of the micronized IBF microparticles was determined by Malvern mastersizer 3000 until we produced the desired size (1–5 µm) and was visualized by scanning electron microscopy (SEM).

#### 2.2.2. Lyophilization

IBF particles were collected immediately after preparation and were frozen at −40 °C for 2 to 3 days, were freeze-dried using a lyophilizer (Alpha 1–4 LD plus, Martin Christ, Osterode, Germany) at −80 °C and were vacuumed at 1.0 mbar for 72 h [14]. Once the drying was completed, the IBF microparticles were stored in glass vials in a desiccator with the presence of silica gel in vacuum at room temperature and 50% relative humidity. To monitor the changes of moisture content, samples were weighed twice a week and data were recorded.

#### 2.2.3. Development and Composition of DPI Formulations by Beads Blending Method

About 2.5% of inhalable sized IBF microparticles were mixed with different concentrations of dispersibility enhancers, such as leucine (2.5–6.25%), MgSt (2.5%) and large carrier lactose (91.25–97.5%), as presented in Table 1. Firstly, lactose was weighed and divided into two portions and one of the portions was placed in a 10 mL glass test tube. Secondly, other materials such as drug and excipients were added to this and 3–4 glass beads (10 mm diameter) were placed on the vial containing 3 glass beads to ensure the ball-milling during mixing. Finally, the remainder of the portion of lactose particles was added and gently shaken by hand for 5–7 min, to reduce the chance of producing any charge or agglomeration among the particles. Thus, the ball-milling helped deagglomerate the agglomerates during the powder blending. Each batch was prepared more than 4 times containing optimized concentrations of micronized IBF, carrier, and excipients with a fixed batch size of 2.0 g. The developed formulations were stored in a desiccator in the presence of silica gel with vacuum conditions at room temperature (20–25 °C, relative humidity as 50–65%) for subsequent experiments. 

#### 2.2.4. Content Homogeneity Test

Content homogeneity was performed to determine the distribution of IBF throughout the DPI formulations. The homogeneity test of the blended DPI formulations was performed not only before all characterization parameters but also before aerosolization studies. The mean content of IBF and the homogeneity variation among the developed DPI formulations were evaluated and presented as % CV (Table 1). Around ten samples from each blended DPI formulations were weighed (20.5 ± 0.5 mg) and dissolved in 50 mL (40% Ethanol, HPLC grade) solution and sonicated for 5 min. The amount of IBF was determined by means of a validated HPLC assay method. This test was accomplished with a mean drug content of 100 ± 5% (Mean ± Standard Deviation) with a coefficient of variation of <5% for all the DPI formulations [1,3,15]. After determining % CV, each formulated powder mixture was manually filled in a hard gelatine capsule shell (Size 3). 

#### 2.2.5. Characterization of the Prepared DPI Formulations

##### Particle Sizing and Size Distribution by Laser Diffraction

Using a laser diffraction technique (Malvern Mastersizer 3000, Malvern Panalytical Ltd, Malvern, United Kingdom), the sizes and size distributions of the milled IBF microparticles were determined. Suspended samples were poured in a small volume in a sample dispersion unit in which the intensity of light scattered as a laser beam passes through a suspended particle sample. All microparticle samples including original and milled before and after lyophilized IBF microparticles were suspended in suspending media. About 5–10 mg of the milled particles from each batch were suspended in 10–15 mL of suspending media and subjected to sonication for 15 min. The sonication helped deagglomerate the agglomerates of the milled particles so that the single particles in the suspensions were available for appropriate size measurement. The sonicated suspensions were added drop wise to the dispersion unit of the Mastersizer until the obscuration bar reached 18 ± 2%. Finally, the size distribution was determined as relative volume density versus size distribution curves. The refractive index for IBF was 1.43 (Malvern Guide, 2007 [16] and for saturated IBF in 0.05% Tween 80 as dispersant was 1.473 (Chemical book, 2017). The particle size and size distribution were determined by the parameters D_10_, D_50_ and D_90_, which represented the particle sizes at 10%, 50% and 90% of the particles smaller than the overall population and mean volume diameter, respectively [14]. The particle size distribution of each sample was determined in triplicate (n = 3). The size distribution span of the micronized IBF was calculated using the following equation:

(1) $$Span=\frac{D_{90}-D_{10}}{D_{50}}$$

##### The Short-Term Stability Studies

The short-term physical stabilities of the developed IBF microparticles were determined in the suspending medium (0.05% Tween 80 solution) over a short period [17]. The developed IBF microparticle size and size distribution were examined at time intervals of 1, 2 and 3 months at room temperature (20–25 °C, relative humidity as 50–65%) using a Malvern Mastersizer 3000. Tests were performed in triplicate (n = 3, Mean ± SD). 

##### Surface Morphology by Scanning Electron Microscopy (SEM)

The surface morphological studies of DPI formulations were investigated using a scanning electron microscope, Tescan Mira3 (Brno, Czech Republic). Using the same machine and Image J software, micronized inhalable particles were visualized. DPI formulations were dusted individually on a metal sample stub with a double-sided adhesive carbon tape attached on its surface. After sprinkling the samples on the top of the adhesive carbon tape, excess particles from the carbon tape surfaces were removed by blowing compressed nitrogen gas. Using a sputter coater, each sample was coated with a conductive layer of gold (Leica EM SCD005 gold coater) with argon gas at 0.5 mbar pressure for 70 to 75 s. The purpose of the gold coating was to prevent surface charging and reduce thermal damage of the particle during imaging at 5 kV. 

##### ATR-FTIR

To determine the compatibility among the API, carrier, and excipients, and to investigate the potential chemical identification and possible molecular interactions, an ATR-FTIR was used to identify each functional group. Fourier Transform Infrared (FTIR) spectra of original, micronized (both wet milled and dry milled) IBF, carrier (lactose), excipients (leucine, MgSt) and DPI formulation were analyzed by a Bruker Alpha-P FTIR equipped with an ATR accessory, which is a single reflection diamond crystal. ATR has an angle of incidence equal to 40 °C with a detector to detect spectra. ATR used with the combination of FTIR allows solid samples to be examined directly without doing any further preparation [4]. A small amount (2–5 mg) of powder sample was placed on the top of the diamond crystal and the high-pressure anvil was gently pressed down towards the sample until it touched the sample surface. The spectra of samples were collected at a resolution of 8 cm^−1^ with 64 scans at wavenumber between 4000–400 cm^−1^. The spectra were analyzed by OPUS analytical software (Bruker Alpha-P FTIR, Version 7.0 or 7.5, Bremen, Germany).

##### X-ray Powder Diffraction (XPRD)

IBF is a crystalline drug molecule and its crystallinity characteristics before and after milling were measured and investigated by XPRD analysis. In this analysis, a smaller average crystalline size (coherently scattering domain size) of a crystalline phase shows broader XPRD peaks [18]. The XPRD patterns of the developed DPI samples were collected in a Debye-Scherrer geometry (capillary transmission mode) using Rigaku^®^ SmartLab equipped with CuKα (λ = 1.5418 Å) sealed X-ray tube operated at 40 kV and 40 mA. The powdered samples were packed into φ 1 mm borosilicate capillaries, which were spun at 15 rpm during data collection. An elliptical primary mirror in CBO-E module was used to suppress CuKβ photons and white radiation background and focus the CuKα X-ray beam to the Hypix3000 detector, which simultaneously collected diffraction signals over 3.81 °2θ. The primary and secondary goniometer radii were 300 mm. Here, two 2.5° Soller slits were used on both the primary and secondary side to reduce axial aberration. An incident slit sized 1 mm was used to illuminate the capillary, the length of which was controlled by a 15 mm heighted mask. On the secondary side, a 6.6 mm anti-scattering slit was placed 50 mm from the capillaries and a 12 mm RS1 slit was placed 185 mm from the capillary and the X-ray diffraction patterns were collected from 3–150° 2θ with a step size of 0.02° at a scan speed of 2.5°/min. XPRD patterns of original, milled microparticles and DPI formulations were compared to identify any chemical or physical alterations in their crystallinity characteristics after preparing the DPI mixtures.

##### Differential Scanning Calorimetry (DSC)

DSC can be defined as the heat flow difference between a sample and a reference material which are subjected to the same temperature and surrounding atmosphere. It provides information about the physical and chemical changes that occur in a sample. The determination of the thermal properties of original, wet milled, dry milled and DPI formulations was carried out by NETZSCH DSC 204F1 Phoenix, Germany. About 6 ± 0.1 mg of each sample was weighed in an aluminum pan and tightly sealed with an aluminum lid (Hermetic pan and lid, USA) to maintain the constant temperature in the pan. An empty sealed aluminum pan and another sealed aluminum pan were used as reference and baseline, respectively. The temperature was set between 20–150 °C at the heating rate of 10 °C/min under continuous purging of nitrogen at 50 mL/min to maintain the appropriate atmosphere in the chamber. All the samples were performed in triplicate. 

##### Thermogravimetric Analysis (TGA)

Not only calorimetric behavior, but the thermal decomposition studies of all samples including original, milled and DPI formulations were determined by thermalgravimetric analysis. TGA is a technique where the mass of a sample is continuously measured as a function of temperature in a controlled atmosphere. All the samples were investigated by a NETZSCH Jupiter high temperature simultaneous analyzer, to determine the decomposition characteristics of the samples. Approximately 20 ± 0.5 mg sample was packed in an aluminum crucible (Al_2_O_3_, 85 µL) with lid. Two empty aluminum pan and lid were used as reference and baseline, respectively. The temperature was set between 25–450 °C at the rate of 10 °C/min increment under dry nitrogen flowing at a 40 mL/min. Data were analyzed by Proteus analysis software (NETZSCH group, Selb, Germany).

##### Powder Flow Property Measurement

Density measurements for milled IBF microparticles along with the original drug were investigated by a tapped density tester (Erweka, Germany; Model no: ERW-SVM101201). The density of IBF particles is 1.110 g/cm^3^ [19,20] and lactose is 1.525 g/cm^3^ (Wikipedia, 2021). The density of the particle can be measured by bulk density (ρ_B_) and tapped density (ρ_T_) calculations. Because of the small number of samples, we used a modified method [21] for determining the density of the powder mixtures. A 10 mL graduated cylinder was fitted with a silicon flat pad (underneath) and a molded adapter (attached to the upper bottom part of cylinder). This 10 mL graduated cylinder was taken as a substitute to the 100 mL graduated cylinder for small mass of powder. A mass (m_0_) of 2 ± 0.2 g of powder each sample (original, micronized and DPI formulation) was weighed and transferred in the 10 mL cylinder. The initial occupied volume of the sample was determined and recorded as (v_0_) and after mechanically tapping (100, 500 and 1250 taps) the same powder mixture, the final tapped volumes were recorded as (v_1_). However, changes in the bulk density (ρ_B_) and tapped density (ρ_T_) were determined by calculating the Carr’s compressibility index, CI and Hausner ratio, HR by the following equations:

(2) $$Carr’s\;Compressibility\;Index,CI=\frac{{(\rho }_{T}-\rho_{B})}{\rho_{T}}\times 100$$

(3) $$Hausner\;Ratio,HR=\frac{\rho_{T}}{\rho_{B}}$$

The flowability measurements of the developed DPI mixtures were determined using an accepted flowability scale for Carr’s compressibility index (CI) and Hausner ratio (HR) [14].

##### Angle of Repose (θ) Measurement for Powder Cohesion and Flowability

One of the important macroscopic parameters used to evaluate the flowability of particles is the angle of repose, θ [22]. The angle of repose measurements were evaluated by the angle formed from the horizontal base of the powders and the edge of the cone-like pile of the same powders. This was a modified version of the USP method [23]. A funnel of 33 mm diameter with orifice 2 mm was used in this process. The height from the beginning of the funnel to the end of the orifice was 55 mm. The funnel was fixed by a stand 3 cm above the petri dish surface. Around 1.0 g ± 0.005 g of powder (original ibuprofen, milled, and DPI formulations) was allowed to flow onto the petri dish to build the cone or pile by pouring the powder through the funnel under the force of gravity. The height of the pile (h) and diameter (d) of the pile base formed by the powder were measured and determined by the following equation:

(4) $$Angle\;of\;repose,\theta ={tan}^{-1}(\frac{2h}{d})$$

##### Ibuprofen Quantification Analysis by HPLC

Ibuprofen was quantified by a validated HPLC method developed in our lab [13] using a HPLC (Agilent Technologies, Model no: HP 1100, serial no: DE33220700, USA) with quad-pumps and HPLC Photo Diode Array (PDA) detector. A Phenomenex C_18_ HPLC column (250 × 4.60 mm, Kinetex^®^ 5µ XB-C18 100A) linked with a C_18_ guard column was used to determine IBF. The wavelength was fixed at 214 nm for IBF. A mixture of acetonitrile, ultra-purified water and orthophosphoric acid (85%) in the ratio of 34:65.95:0.05 (*v*/*v*) was used as a mobile phase. The flow rate of the mobile phase was set at 1.2 mL/min. Using an autosampler, 20 µL solution was injected. The retention time of IBF was found to be between 7–8 min. The limit of detection (LOD) of IBF in the solution was 0.05 µg/mL. The stock solution of the drug was prepared by weighing 5 mg of original IBF into 100 mL of 40% ethanol solution as solvent and this was sonicated for 15 min until complete dissolution of IBF in the solvent occurred. Using the same solvent, the stock solution (50 µg/mL) was diluted to a series of different concentrations between 0.25 µg/mL and 20 µg/mL to obtain the data for constructing the calibration plot.

##### In Vitro Aerosolization Performance by TSI

The in vitro dispersibility of the prepared IBF microparticles was investigated by a Twin stage impinger (TSI, Copley Scientific, Nottingham, UK). Approximately, 33 ± 2 mg of each formulation was filled in a hard gelatine capsule shell (size #3) which was inserted into the DPI device Breezhaler^®^ (Novartis Pharmaceuticals, Basel, Switzerland). The concentration of drug in each capsule ranged between 775 to 875 µg. The upper stage of the TSI represented as stage 1 (S1) was filled with 7 mL solvent (40% ethanol solution in water) and the lower stage (S2) was filled with 30 mL of the same solvent. The actuation of the formulation was carried out by the vacuum pump (ERWEKA GmbH, Hessen, Germany) through the mouthpiece using a calibrated flow meter (Fisher and Porter Ltd, Workington, UK). For each capsule, actuation through the vacuum pump was operated for 5 sat 60 ± 5 L/min. Samples were collected individually by rinsing the device; S1 and S2 compartments and IBF were determined by a validated HPLC method as explained before. The aerosolization performance of the prepared formulations was characterized by common parameters including recovered dose (RD), emitted dose (ED), fine particle fraction (FPF) and fine particle dose (FPD). RD can be defined as the total amount of IBF accumulated in the Breezhaler^®^ (BH), S1 and S2. Breezhaler^®^ contains capsule, mouthpiece and the device. ED can be defined as the % of RD delivered from Breezhaler^®^. FPF can be described as the % of RD deposited in the S2 of the TSI and finally FPD can be described as the mass of IBF deposited in S2. The results of IBF aerosolization behavior are provided in Table 4. Equations for RD, ED, FPF and FPD are as follows:

(5) RD=BH+S1+S2

(6) $$ED=\frac{\left(S1+S2\right)}{RD}\times 100$$

(7) $$FPF=\frac{S2}{RD}\times 100$$

FPD = mass of particles deposited in S2 of TSI

##### Saturated Solubility Studies in PBS

To evaluate the solubility of original and micronized IBF, a saturated solution was prepared by transferring 50 mg of the active drug (IBF) in a 50 mL centrifuge tube; 5 mL phosphate buffer saline was added and it was stirred at 600 rpm for 24 h at 22–25 °C temperature by a magnetic stirrer [13]. pH was measured by a calibrated pH meter (TPS AQUA, Queensland, Australia) and adjusted to 7.4. Accordingly, 2 g of each DPI formulation mixture was transferred to a 50 mL centrifuge tube to determine the saturation solubility of the drug in the presence of excipients. The supernatant was collected after centrifuging the solutions of each sample at 5000 rpm for 30 min, filtered by a 0.22 µm syringe filter. The sample was diluted 10 times and was analyzed by HPLC. Each experiment was performed in triplicate. The standard stock solution of IBF was prepared by weighing 5 mg of original IBF into 100 mL PBS followed by sonication for 15 min until it completely dissolved in the solvent. Using PBS solution, the prepared stock solution was diluted for HPLC analysis as demonstrated in “Ibuprofen Quantification Analysis by HPLC”. The concentrations of IBF were measured and we determined the saturated solubility of the IBF in PBS after 24 h. 

##### In Vitro Dissolution of the IBF from the Formulations

Using a USP Dissolution Apparatus 2 or paddle method (ERWEKA, Germany), the dissolution performances of all the DPI formulations and the milled IBF microparticles at 100 rpm at 37 ± 0.5 °C were analyzed [13]. Accurately weighed micronized IBF (12.5 mg) powder and the prepared DPI formulations equivalent to 12.5 mg were added to the dissolution media (PBS, pH 7.4). Each sample was placed in 900 mL of dissolution media. After starting the dissolution process, 5 mL of samples were withdrawn at a predefined time interval of 2.5, 5, 10, 15, 20, 30, 60, 90, and 120 min, respectively. Immediately after withdrawing samples, 5 mL of fresh PBS solution was added to the dissolution media to maintain a constant volume during the dissolution process. The collected samples were filtered through a syringe filter (0.22 µm) and the amount of IBF was determined by a validated HPLC method. Each experiment was performed in triplicate (n = 3, Mean ± SD). 

##### Statistical Analysis and Graphical Representation

Using Microsoft^®^ Excel^®^ (16.0.14026.20294), all statistical analysis was carried out by applying one-way analysis of variance (ANOVA). To ascertain significant differences, *p* < 0.05 was used. All the graphics were created using Origin software (OriginLab Corporation, Massachusetts, USA). 

## 3. Results and Discussion

### 3.1. Particle Sizing and Size Distribution by Laser Diffraction 

The particle size and size distribution of the developed inhalable IBF microparticles were obtained by laser diffraction as presented in Table 2. It is clearly seen that the original ibuprofen particle size (D_50_, 71.3 µm) is significantly reduced after wet milling and dry milling processes. Overall, D_50_ values of all IBF microparticles before and after freeze-drying were less than 5 µm. Among all micronized samples, IBF containing leucine (5–6.25%) showed the lowest particle size (D_50_, 1.71 µm). The obtained D_[4,3]_ values and corresponding span are very similar before and after freeze-drying the milled samples, which indicated the limited agglomeration tendency of the particles. This outcome suggested that the original ibuprofen particles were successfully micronized by the wet milling process and produced inhalable particles (<5 µm).

### 3.2. Short-Term Stability Studies

**Figure 1:**
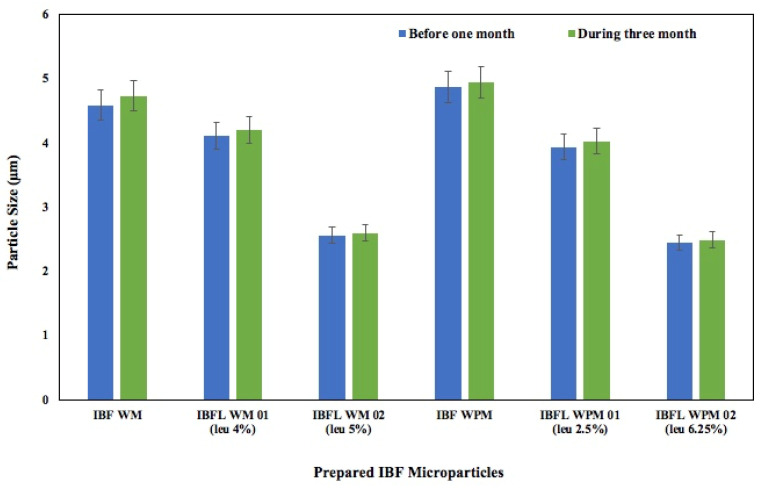
Prepared IBF particle sizes before and after one month of short storage (Mean ± SD, n = 3).

The short-term stability studies after one month revealed that the size of the freeze-dried prepared IBF microparticles slightly increased. Likewise, wet milled IBF microparticles slightly increased in size from 1.71 to 1.78 ± 0.14 µm, which is still an inhalable size and acceptable for DPI formulation preparations. However, no significant (*p* = 0.565) changes in the size of either wet milled or wet paste milled IBF microparticles were noted after one month of storage in silica gel desiccator. However, it was observed that the particle size containing 2–6.25% leucine did not increase (*p* > 0.05) during the storage period (Figure 1). The dispersibility enhancer leucine might have helped reduce the agglomeration of the micronized particles. Although ICH guidelines indicated a minimum of 6 months stability, we carried out a stability study for 3 months following some published research which demonstrated stability studies only for one month [17] Furthermore, the inhaled DPI products are mostly used within 3 months after opening the devices. Thus, our short-term stability studies are considered as a valid stability study as per published research. However, a long-term stability study may be warranted to understand the agglomeration behavior of the formed particles.

### 3.3. Surface Morphology by Scanning Electron Microscopy (SEM)

**Figure 2:**
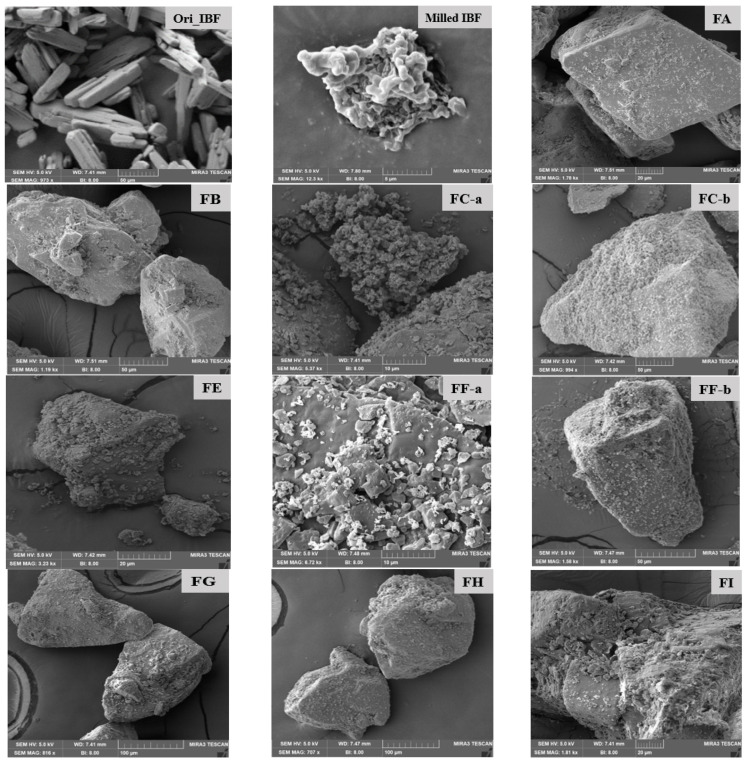

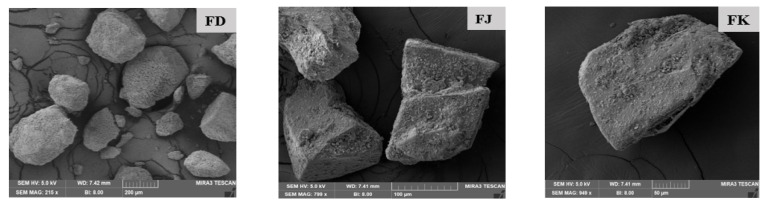
SEM images of original ibuprofen, milled IBF, and developed DPI formulations (FA, FB, FC-a, FC-b, FE, FF-a, FF-b, FG, FH, FI, FD, FJ and FK).

The morphological structure of original ibuprofen, micronized IBF and developed DPI formulations was obtained by SEM imaging. The original ibuprofen particles appeared as needle-like shapes with sharp edges and slightly rough surface (Figure 2: Original IBF). After milling, inhalable sized particles (<5 µm) were small with less irregularities (Figure 2: milled microparticle) along with spherical-shaped smaller agglomerates, which were found to be smaller than the original drug particles. SEM images also confirmed the drug particle size between 0.4 to 5 µm. However, DPI formulations appeared as a physical mixture of large carrier lactose in which micronized IBF particles were attached to the surface of large carrier lactose particles (Figure 2).

The SEM images of unmilled original IBF, milled IBF, and prepared DPI formulation mixtures (Figure 2: FA, FB, FC-a, FC-b, FE, FF-a, FF-b, FG, FH, FI, FD, FJ and FK) are presented in Figure 2. It is clearly visible that the physical mixtures were formed by mixing milled particles with large lactose (≤ 200 µm) where micronized drug particles adhered on the surfaces of the carriers. The formulation FC-a and FC-b illustrated more adhered microparticles as small aggregates than those of the formulations of FA, FB, and FF-a, where more leucine (4–6.25%) was added. This indicated that many small particles of leucine and the drug homogenously adhered to the surface of lactose because of the hydrophilic nature of the high concentration of leucine (4–6.25%) in the formulations. However, formulations FD, FK, and FJ were developed by dry milled IBF microparticles, which appeared as a complex mixture of aggregates adhered on the surface of irregularly shaped large carrier lactose (Figure 2: FD, FJ, and FK). Formulation FK showed less attachment of microparticles, which indicated the absence of additive leucine (Figure 2: FK). However, formulation FJ (Figure 2: FJ) had more aggregates adhered on the surface of lactose than other dry milled formulations, as this formulation contains both leucine and MgSt.

### 3.4. ATR-FTIR Spectral Analysis

**Figure 3:**
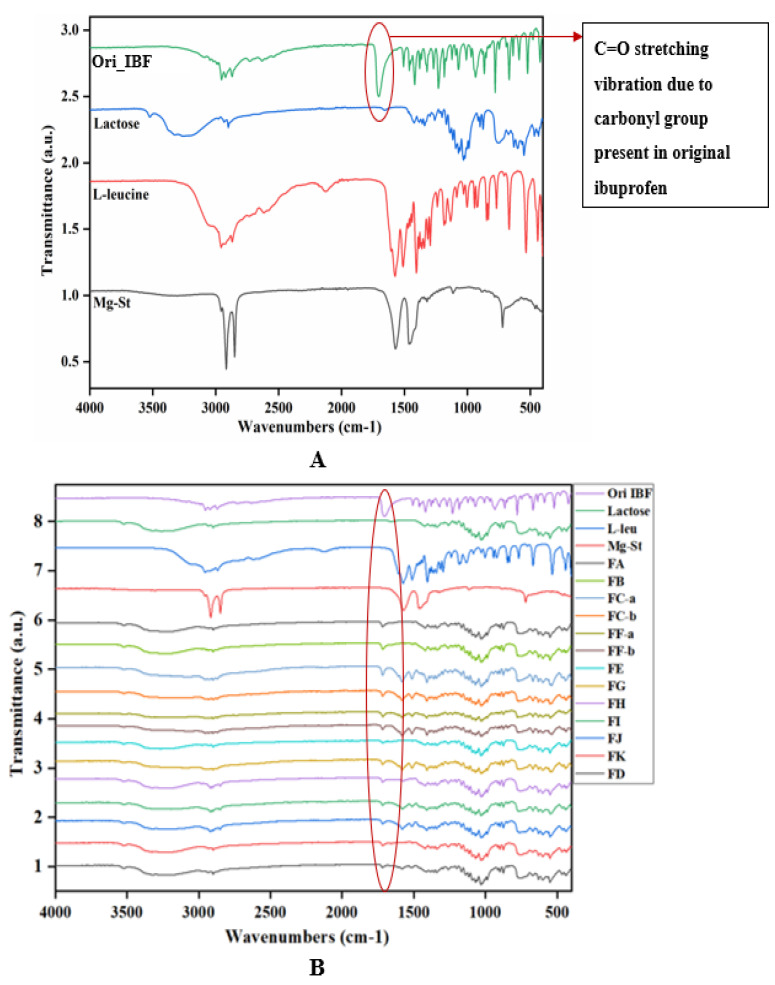
(**A**) Individual FTIR spectra of original ibuprofen, lactose, leucine and MgSt. (**B**) Subtracted FTIR spectra of prepared DPI formulation powder mixtures.

The FTIR spectra of the original drug, milled IBF and prepared DPI formulations containing drug particles, carrier lactose, excipients leucine and MgSt in different ratios are given in Figure 2. The original ibuprofen showed strong and sharp characteristic absorption peaks at 1707 cm^−1^, 2957 cm^−1^, and 779 cm^−1^ due to the presence of the carbonyl group from propionic acid (C=O stretching vibration), CH_3_ asymmetric stretching for OH stretching vibration, and CH_2_ rocking vibration of isobutyl moiety from aromatic benzene ring, respectively [24,25], which match previous reports. After preparing the DPI formulations with IBF microparticles and excipients (Figure 3B), no major significant change in characteristic absorption peaks were detected and only minor shifting was observed from 1712 cm^−1^ to 1718 cm^−1^ in the DPI formulation mixtures. All formulations illustrated characteristic absorption peaks ranging from 1718 cm^−1^–1712.79 cm^−1^ corresponding to the characteristic absorption peaks of the original drug [21]. DPI powder mixtures FA, FB, and FK from Figure 2 contain milled IBF with lactose carrier only. Due to the presence of the -OH bond in the lactose, the characteristic absorption peak of IBF at C=O stretching vibration slightly shifted from 1707–1714.34 cm^−1^ but the result indicated no chemical reaction between IBF and lactose as there was no observation of other peaks. DPI mixtures FC-a, FC-b, FE, FF-a, FF-b, FG, and FD from Figure 2 showed that the characteristic absorption peak of IBF at C=O stretching vibration slightly shifted from 1707–1717.43 cm^−1^ but the result still indicated no chemical reaction observed between drug and excipients. Furthermore, DPI mixtures FH, FI and FJ from Figure 2 demonstrated that the characteristic peak of IBF at C=O stretching vibration slightly shifted from 1707–1716.53 cm^−1^ but the result indicated no chemical interaction observed among IBF and excipients as the result is within the range of the original drug, indicating that no chemical reaction was observed among 2.5% IBF with 92.5–97.5% lactose, 2.5–6.25% leucine, and 2.5% MgSt.

To confirm the existence or non-existence of any kind of chemical reaction or interaction among IBF with excipients from prepared DPI formulations, the characteristic peaks of the drugs were subtracted (Figure 3B) from all formulations. Figure 3A clearly shows that original IBF only showed characteristic peaks between 1718–1700 cm^−1^ due to the presence of C=O stretching vibrations from the carbonyl group. No other materials had absorption peaks within this area (Figure 3A). However, the subtracted results (Figure 3B) showed no remarkable change (red circle) observed among IBF microparticles and excipients. A minor shift in the absorption peak of original IBF occurred due to the presence of possible -OH bonds in lactose and leucine [1,4]. Thus, no specific chemical changes or interaction were noticed among the drug and excipients from the developed DPI formulations. Therefore, the excipients used in all formulations are compatible with the active drug.

### 3.5. XPRD Analysis

**Figure 4:**
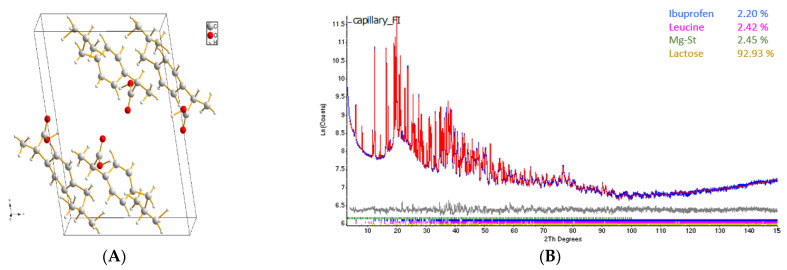
(**A**) Refined ibuprofen molecules from PXRD analysis and (**B**) quantitative phase analysis of DPI formulation FI, which was formulated with 2.5% IBF WPM, 2.5% leucine, 2.5% MgSt and 92.5% lactose carrier.

The quantitative phase analysis of the prepared IBF was carried out using the refined IBF crystal structure presented in Figure 4A and according to the published literature [3]. The percent amounts of each phase are presented on the top right corner of Figure 4B, representing the DPI formulation FI which consisted of 2.5% wet milled IBF microparticles, 2.5% leucine, 2.5% MgSt and 92.5% lactose particles (Table 1). The XRD patterns of original and both wet and dry milled IBF microparticles showed no changes in the crystallinity of IBF confirmed by their same peak positions and relative intensities, supported by the ICDD PDF database (# 02-097-4022). Additionally, the broader XRD diffraction peaks of the milled IBF compared to the original IBF reflected that the crystallinity size was effectively reduced by the milling processes. The confirmed crystal structure of the carrier lactose monohydrate, dispersibility enhancer leucine and the calibrated PONKCS model of flow promoter MgSt in the DPI formulation are supported by other research [1,3]. The quantitative phase analysis exposing the presence of excipients with the IBF microparticles was further quantified in % (Figure 4B). The theoretical % of the excipients represented the % amount of carbon atoms in each molecule as determined by their molar compositions, which indicated the existence of lubricating agent MgSt in the DPI formulation mixture. The result confirmed both excipients grater surface coverage by quantitative phase analysis.

### 3.6. DSC Analysis

**Figure 5:**
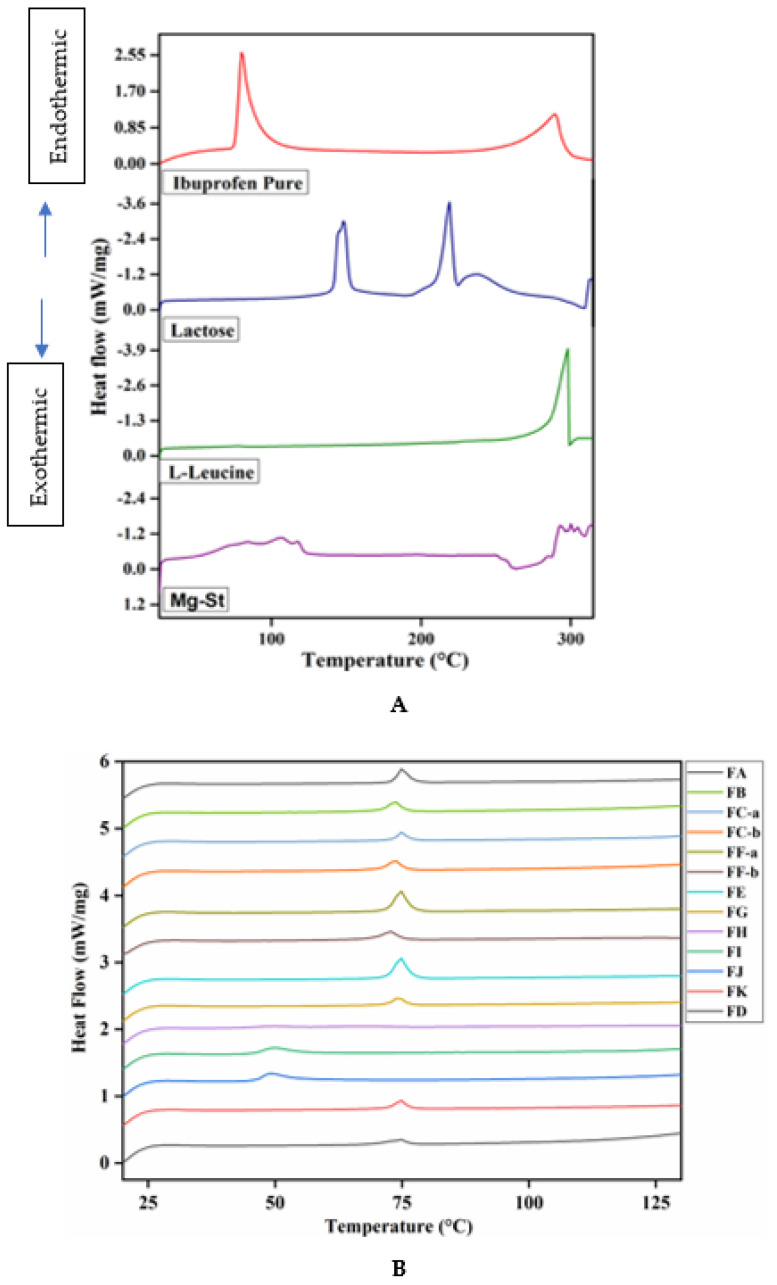
(**A**) Stacked DSC thermograms of original IBF, lactose, leucine, and MgSt. (**B**) Prepared DPI formulation mixtures. (Mean ± SD, n = 3).

The melting point of IBF is 75–78.5 °C or 167–172 °F [26]. Here, Figure 5A illustrated individual sharp endothermic peaks as melting points of the drug, carrier, and excipients. Figure 5B shows the melting point of the prepared DPI formulation mixtures. Figure 5A illustrated a sharp endothermic peak as the melting point for the original drug at 76–77 °C (within ± 1 °C). The previous literature suggests that the crystalline state of the original drug exhibited melting points within this range [11]. However, the melting points of the carrier and excipients are different from the original drugs presented in Figure 5A. This result indicated that IBF can be easily identified by DSC thermograms in the mixture. The endothermic peak between 75–76.7 °C, regarded as the melting point of original drug and the milled IBF microparticles with leucine, MgSt and lactose, are presented in Figure 5B for FA, FB, and FE. No other endothermic peak or phase transition was observed in these samples and this indicates that no thermal changes, chemical interactions or degradation occurred between wet milled micronized drugs and lactose particles. Moreover, formulation FC-a, FC-b, FF-a, and FF-b were prepared by mixing wet milled micronized IBF particles (2.5%) with 2.5–6.25% leucine and 91.25–93.5% lactose. The melting point for these formulations was found to be within 75.5–76.7 °C, which indicated no changes in the thermal properties of IBF in the developed formulations.

However, DPI formulations FG, FH and FI formulated by 2.5% wet milled IBF microparticles with 2.5–5% leucine, 2.5% MgSt and 92.5–95% lactose showed endothermic broader peaks between 62.8–76.5 °C, corresponding to the melting point of amorphous IBF polymorphs that developed due to the milling process [11]. Nonetheless, formulations FH and FI showed slight early exothermic peaks of IBF at 62.8 °C and 63.2 °C, respectively, due to the presence of MgSt. However, the melting points of formulations FH and FI were significantly reduced, which somewhat indicated that the stability of the original drug slightly degraded due to the presence of MgSt compared with the formulation mixtures without MgSt i.e., FA, FC-a, and FF-b. The change in the melting points demonstrated the fast degradation of the drug exposed to high temperature [3]. The DPI formulations FD, FK and FJ (Figure 5B) prepared by mixing 2.5% dry milled IBF microparticles with 2.5% leucine, 2.5% MgSt and 92.5–97.5% lactose showed endothermic peaks between 58.6–77.5 °C, which indicated the melting point of IBF. Formulations FH, FI and FJ containing 2.5% MgSt showed faster melting points than the original IBF particles. This change in the melting point could be due to the fast degradation of the IBF due to the presence of MgSt when exposed to high temperature (20–600 °C) [3]. Therefore, stacked DSC thermograms of DPI mixtures from Figure 5B clearly show most of the formulations’ melting point ranges between 75–77.5 °C; however, formulations FH, FI and FJ (Figure 5B) showed earlier melting points between 58.6–63.2 °C due to the presence of MgSt (Table 1). As a result, the DSC thermograms confirmed that the presence of MgSt slightly interfered with the melting properties of the drug IBF, but leucine did not interfere with the IBF microparticles in the DPI formulation mixtures.

### 3.7. TGA Analysis

**Figure 6:**
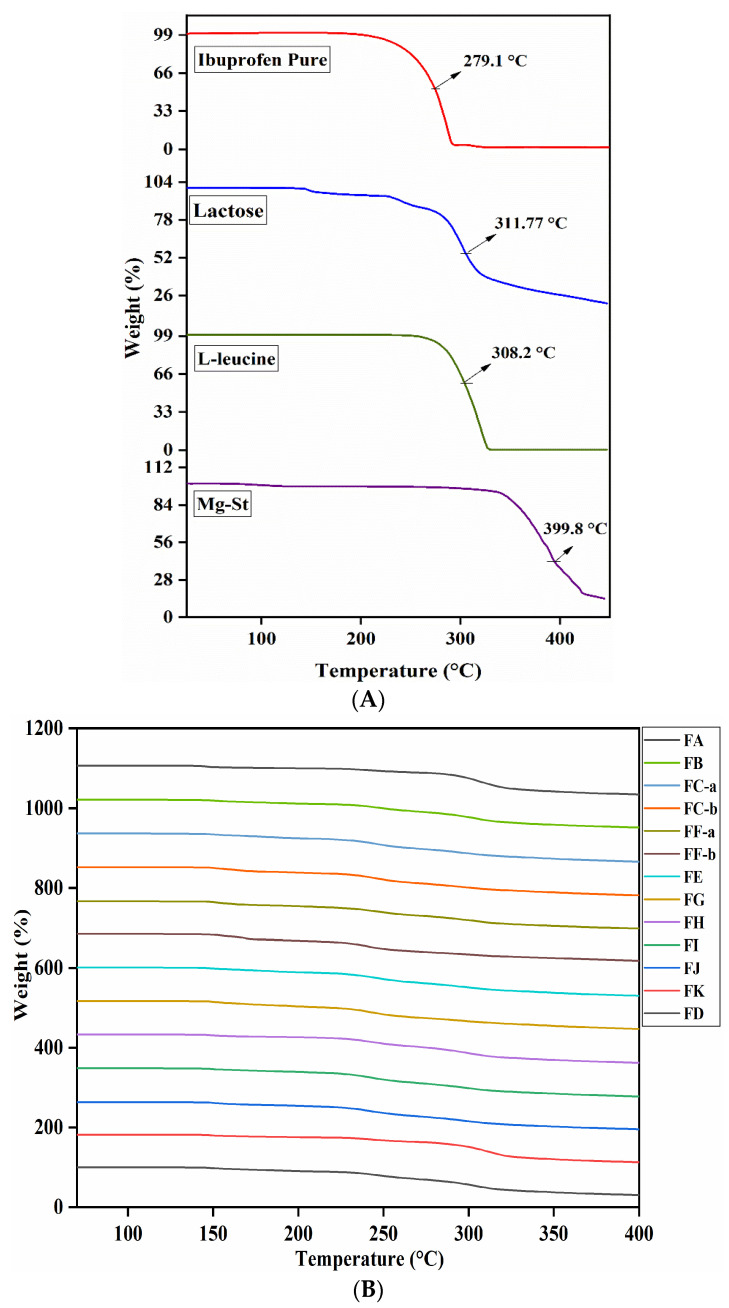
Stacked TGA thermograms of (**A**) raw materials, (**B**) developed DPI formulations (n = 3, Mean ± SD).

The TGA thermograms of raw materials and the developed DPI formulation mixtures with different concentrated excipients are illustrated in Figure 6. The thermogram of original IBF shown in Figure 6A presents three substantial characteristic weight loss occurrences starting from 120–148 °C due to the loss of surface water. The second mass loss noticed between 150–250 °C is due to the loss of the water of crystallization [27]. Finally, the third weight loss occurred when the temperature increased up to 450 °C. Hence, 50% weight loss was observed within 250–290 °C followed by the decomposition of the samples. However, TGA thermograms of lactose, leucine and MgSt had similar weight loss structure and decomposition behavior, but this was not the same as IBF (Figure 6A). These results indicated that IBF can be easily detected from the DPI formulation mixtures. Figure 6B presents the DSC thermograms of formulations FA, FB, and FE, which contain 95–97.5% lactose in the mixtures. The 50% weight loss of these formulations was found to be at 306.4–306.7 °C, which significantly matched the decomposition behavior of the lactose particles (Figure 6B—lactose). Original and milled IBF microparticles showed 50% mass loss after 270 °C and decomposition behavior after temperature rises to 300 °C. So, no changes occurred in the formulations which contained 91.25–97.5% lactose as all formulations illustrated mass loss the same as lactose. This result indicated that no chemical incompatibilities occurred among the drug, lactose and leucine in the formulations FA, FB, and FE (Figure 6B; FA, FB, and FE). TGA thermograms of formulations FC-a, FC-b, FF-a, and FF-b (Figure 6B) containing 2.5% wet milled IBF with 2.5–6.25% leucine and 91.25–93.5% lactose showed similar thermal characteristics as 50% mass loss occurred at 310°C–312 °C. Thus, it can be confirmed that there were no changes observed in the thermal properties of the drug IBF, which indicated no chemical incompatibilities among the formulation components i.e., IBF, leucine, and lactose. However, TGA thermograms of formulations FG, FH and FI, formulated by 2.5% wet paste milled IBF with 2.5–5% leucine, 2.5% MgSt and 92.5–95% lactose, illustrated characteristic weight loss the same as previously described formulations. Even though 50% of mass loss was observed within 302.8 °C–310.3 °C followed by the weight loss of other formulations, FH and FI showed decomposition within 302.8–303.7 °C, which might be due to the presence of MgSt. The thermograms of formulations FK, FD and FJ prepared by 2.5% dry milled IBF with 2.5% leucine, 2.5% MgSt and 92.5–97.5% lactose showed 50% weight loss at 306–321.9 °C. However, FJ mass loss (50%) occurred at 321.9 °C, which might be due to the presence of MgSt. However, this difference confirms the slightly delayed weight loss which occurred due to the presence of MgSt, as it individually produced mass loss after 350 °C and independently showed 50% decomposition at 399.8 °C (Figure 6A; MgSt).

Therefore, the TGA analysis indicated no chemical incompatibilities among the powder mixtures containing IBF, leucine, MgSt, and lactose. This result concludes that MgSt is a compatible excipient with the drug IBF. As a result, the characteristic behavior of the developed IBF microparticles and prepared DPI formulations indicated the thermal stability in solid form in the presence of leucine and MgSt.

### 3.8. Powder Density and Flow Property Measurement

The bulk density and the tapped density of the developed DPI formulations were evaluated to understand, identify, and confirm the developed DPI formulations with inhalable properties and better flowability behavior. Table 3 presents the results on flowability properties of the developed DPI formulation mixtures.

The CI and HR were measured to determine the flowability of the DPI mixtures followed by bulk density and tapped density measurements. The flowability behavior was examined using an accepted measuring scale of CI and HR, which give an estimation of the flow property [11]. When the CI value is ≤10%, it indicates an excellent or very free flowability whereas a CI of >25% is considered as poor flowability of the powder. However, the HR value between 1–1.11 is considered as excellent flowability behavior and a HR value >1.34 indicates poor flow properties [28] because of dominant cohesive forces among the particles. Formulations FC-b, FF-a, FF-b, FG, and FH showed excellent flowability among all DPI formulation mixtures, in which the CI values ranged from 9.27–10.5% and HR values ranged from 1.10–1.11; these formulations contained 2.5% wet paste milled IBF with 4–6.25% leucine, 2.5% MgSt and 91.25–95% lactose and the results indicated that both parameters showed very free flowing properties of the powder mixture (Table 3). Moreover, formulations FA, FB, FC-a, FE, and FI showed good flowability with CI values ranging from 12.2–14.5%. These were formulated by wet milled IBF microparticles with 2.5% leucine as a flowability enhancing agent along with 92.5–97.5% lactose. The HR values of these formulation ranged from 1.14–1.17, indicating good powder flowability. Although formulations FD, FJ and FK, consisting of milled IBF microparticles, whose flowability was previously very poor due to the particle–particle cohesion which occurred during the dry milling process, DPI formulations containing excipients showed good flowability (CI as 13.7–14.8% and HR as 1.15–1.17) due to the presence of lactose, leucine, and MgSt. Formulation FK had CI and HR values of 19.1% and 1.24, respectively, which can be considered as fair flowability due to the absence of leucine and MgSt in the powder mixture. As a result, it seems that the formulation containing the maximum amount of leucine (5–6.25%) helped improve the powder flowability compared to the other mixtures due to the presence of anti-adherent and lubricating agents. This indicated that the concentration of leucine above 4% in the DPI formulation would lead to excellent flowability. Here in this study, the improved flow property of the milled IBF with leucine and MgSt might be achieved in different ways. The excipient leucine oriented itself in a way around the IBF so that hydrophobic domain of leucine was inclined towards the hydrophobic IBF and hydrophilic domain towards lactose, which changed the particle interactions in the formulations and enhanced the flowability [8,29]. In another study, physically mixing MgSt with lactose and salmeterol xinafoate modified the particle interactions from IBF–lactose to IBF–MgSt owing to the hydrophobic nature, which was responsible for the enhanced particle detachment from the carrier and enhanced aerosolization [30].

Table 3 summarizes the angle of repose (θ) values of all developed DPI formulations and the effect of excipients and their concentration on the formulations. The θ value has an important role in powder flowability properties, and when the θ value is 25–30° it is considered as a very free flowing powder mixture. However, if the θ value is in between 36–40°, it indicates fair flowability behavior. Formulations containing 2.5–6.25% leucine showed excellent flowability (θ, 27–30°, Table 3: FC-b, FF-a, FF-b, FG, and FH). Although formulations FA, FB, FC-a, FE, and FI showed good flowability (θ32–35°), FJ showed improved flowability from very poor to good due to the mixture of 2.5% leucine and 2.5% MgSt, since leucine has antiadhesive properties in several cases [21,31]. However, formulation FK did not show much improvement as it contained only carrier particle lactose and had no flowability-enhancing leucine or lubrication agent MgSt. As a result, leucine played a remarkable effect on the powder flow.

### 3.9. In Vitro Aerosolization Performance by TSI

The in vitro aerosolization performances of the developed DPI formulations are presented in Table 4. The ED of all formulations ranged between 79.2–90.3%, which indicated that most powders were efficiently emitted from the device as demonstrated by previous research [1,4], due to the inhalable sized IBF produced by the wet milling method. The formulation mixture of original IBF showed poor ED (16.9 ± 1.5 %) because of the large IBF particles (71.3 ± 6.96 µm), which were not emitted from the device. The FPFs of IBF from wet milled formulations were 6.1–37.1% (Table 5: Formulation FA-FI), whereas FPFs of IBF from dry milled particles were 6.1–15.7% (Table 5: Formulation FD, FJ, FK). The reason that particle–particle cohesion occurred could be due to surface charges or high surface free energy produced during the dry milling process, which reduced the flowability leading to poor aerosolization [32]. The addition of large carrier particle lactose to the formulations FA and FB led to the FPF successively increasing to 15.9%, which is much better as compared to the lactose free formulations. This is due to the presence of lactose, which helped improve flow properties [3] by reducing the particle–particle cohesion. forces. Additionally, the presence of large lactose enhanced the deagglomeration process of the milled IBF in the formulations [33,34,35].

Leucine is a well-known dispersibility-enhancing agent commonly used in DPI formulations [3,8,18,36]. In this study, we used the dispersibility enhancer leucine and MgSt during the milling process to understand the aerosolization properties of all formulations and leucine was found to be a superior excipient over MgSt. The addition of 2.5–6.25% leucine mostly increased the aerosolization performances of the DPI powder mixtures; however, formulations containing 4–6.25% leucine showed significantly (*p* < 0.05) higher FPF (25.3–37.1%) compared to those of formulations without leucine or lower amounts of leucine. It was clearly remarkable that the presence of 6.25% leucine showed the highest FPF (37.1%) compared to other formulations. With regards to the impact of lubricating and dispersibility-enhancing agent MgSt, 2.5% MgSt was added to the formulations FH, FI, and FJ, which showed medium levels of FPF ranging between 16.5–25%. Among these three formulations, FI showed relatively better FPF (24.8%) compared to FH and FJ, due to the presence of 2.5% leucine (Table 4: FI). The lubricating agent MgSt is extremely hydrophobic and might not properly distribute among the powder mixtures containing large amounts of hydrophilic elements, and this led to the poor flow properties of the formulations. The dry milled formulations FD, FJ and FK showed poor FPFs (6.8–17.1%) owing to the inter-particulate cohesion occurring among the particles during the dry milling process [1,4]. Here, a few formulations were developed by mixing both leucine and MgSt. For example, FI and FJ contained 2.5% leucine with 2.5% MgSt and formulation FI showed better FPF (17.1%) compared to others (FD and FK). Hence, it can be confirmed that 6.25% leucine helped increase the flowability and dispersibility of the IBF microparticles and improved the aerosolization performance by reducing the drug particle–particle cohesion forces, improving the easy detachment of IBF from the carrier surfaces. With regard to the enhanced FPFs of the formulations containing leucine, this could be due to the orientation of amphiphilic leucine on the milled IBF surfaces. The IBF particles were surrounded by leucine, which was distributed on the surface according to the interactions governed by the amphiphilic nature and which might change the IBF surface property [37]. In another study, physically mixing lactose and salmeterol xinafoate with MgSt (up to 10%), modified the particle interactions from drug–lactose to drug–MgSt owing to the hydrophobic nature; this surface mechanism was responsible for the enhanced drug particle detachment from carrier surface due to lower interaction forces, and enhanced the aerosolization [30]. In our study, MgSt might have interacted with hydrophobic IBF and enhanced the aerosolization. The hydrophobic and hydrophilic domains of leucine could be oriented in a balanced way in the formulations so that the hydrophilic part is inclined towards the hydrophilic surface of lactose and the hydrophilic part oriented towards the hydrophobic surface of IBF in the mixtures [11]. Overall, these changes in the formulation could be responsible for the easy detachment of IBF particles or agglomerates from the surface of large carriers and enhanced the aerosolization properties.

### 3.10. Saturated Solubility Studies

The saturated solubility of the original and milled IBF and developed DPI formulations were evaluated to check which excipient led to the highest saturated concentration of ibuprofen. Table 5 shows that the maximum saturated solubilities of IBF occurred in wet milled IBF microparticles, with values of 159.1 ± 0.1 µg/mL in 24 h, and this is significantly higher (*p* < 0.5) than the original and dry milled IBF. This must because micro sized particles developed during the wet milling homogenization process using 0.1% Tween 80 solution as solvent, which enhanced the solubility of the drug. However, dry milled and original ibuprofen exhibited saturated solubility values of 149.0 ± 0.1 µg/mL and 147.4 ± 0.8 µg/mL, respectively. This result indicated that the size of the original drug particles was larger than wet milled and dry milled particles and that is why the solvent drug particle interaction was limited, which resulted in slightly lower drug solubility than milled drug particles.

The presence of 2.5% MgSt in the selected DPI formulations produced the highest solubility (252.8 ± 0.6 µg/mL) of IBF compared to unmilled IBF (147.4 ± 1.6 µg/mL). Based on the experimental results, DPI formulations FA and FB showed solubility values of about 157 ± 1.10 µg/mL. This result is quite similar to the wet milled micronized IBF. Moreover, DPI formulations FC-a, FC-b, FF-a, FF-b and FG showed enhanced solubility values of IBF ranging from 162 to 167.5 µg/mL. This could be due to the 2.5–6.25% leucine present in these formulations, which may form co-amorphous dispersion. Leucine is an essential amino acid, which has been found to improve solubility [38]. Surprisingly, FH, FI and FJ showed the highest solubility of IBF (247.2–252.8 µg/mL) among all formulations due to the presence of 2.5% MgSt. While MgSt disperses in basic conditions (PBS, pH 7.4) at a certain temperature and humidity, MgO from the MgSt reacts with carboxyl acid group from IBF, which might cause an acid–base reaction to form magnesium salt of ibuprofen [39]. This result significantly increased the drug solubility (at 24 h of mixing), which could be attributed to the improvement of the drug particle dispersion which helps in drug solubilization [40]. However, MgSt with impurities such as MgO could create an alkaline environment at a specific temperature and humidity, which can cause drug degradation that can be determined by DSC or TGA analysis. Furthermore, FD and FK (Table 5) showed that the solubility of dry milled IBF microparticles is like that of the original IBF. This could be due to the particle–particle cohesion, which caused the formation of agglomeration during dry milling. Conclusively, size reduction and mixing with excipients helped to increase the saturated solubility of ibuprofen, which demonstrates that the wet milled IBF microparticles showed improved and better results than the dry milled IBF microparticles.

### 3.11. In Vitro Dissolution of Ibuprofen

**Figure 7:**
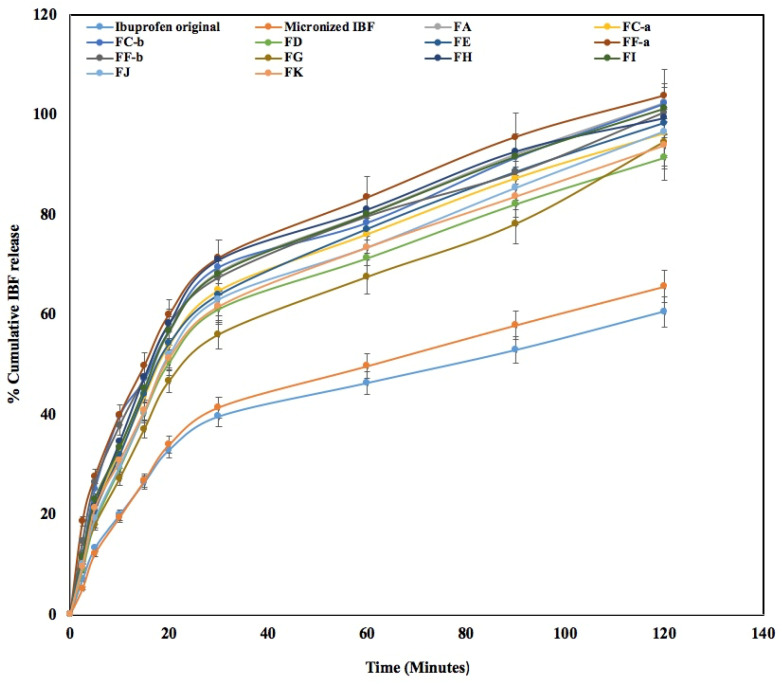
In vitro drug release of original, micronized and DPI formulations by Dissolution study (Mean ± SD, n = 3).

The dissolution studies of DPI formulations were compared with the original drug and micronized drug and are presented in Figure 7, which demonstrates that the dissolution rates of the original IBF were much slower than those of milled IBF and all powder mixtures. However, the wet milled IBF microparticles showed better drug dissolution than the original drug particles (Figure 7: Original and milled IBF). This result indicated that the size of the milled particle is smaller than the original drug and, therefore, the smaller particles interacted more with the solvent and increased the dissolution due to large surface areas. The developed DPI formulations showed faster drug release due to the presence of carrier and excipients. Figure 7 shows that the formulations FF-a, FF-b and FC-b showed faster drug release from the beginning (2.5 min) of the dissolution study with values of 18.6%, 14.7% and 12.2%, respectively, due to the presence of leucine (4–6.25%), mixed with 2.5% of milled drug particle and carrier lactose (91.25–92.5%). This result indicated that the dissolution rate of the developed formulations was significantly faster due to the presence of micronized IBF particles in the presence of amphiphilic leucine and hydrophilic lactose. However, formulations FC-a, FE and FG showed relatively slower drug release due to the lower concentration of leucine used (2.5%). Interestingly, formulations FH, FI and FJ showed slightly faster drug release 80–85 % at 60 min due to the presence of MgSt (2.5%). While MgSt is dispersed in basic conditions such as PBS, faster release or increase in the drug solubility at 24 h could be attributed to improvements in the drug particle dispersion which helped in solubilizing the drug [40]. MgSt might play an important role in enhancing the solubility and dissolution of IBF particles by forming a complex mixture of the drug, such as the Mg salt of IBF that dissociates in the solvent and improves the solubilization [39]. Additionally, faster drug release could be due to the presence of both excipients which reduced the particle–particle cohesion and increased the drug–solvent interactions, thus enhancing the solubility of IBF.

Formulations FA and FK containing 2.5% micronized drug with 97.2% lactose carrier exhibited IBF dissolution of 100% and 93.8%, respectively, in 2 h. Lactose is a disaccharide, which consists of β-D-galactose and β-D-glucose, which contain many hydrophilic hydroxyl groups. Research suggests that hydroxyl groups enhance the dissolution rate of poorly soluble drugs by increasing the drug–solvent interactions [11]. Additionally, the presence of leucine in phosphate buffer significantly improved the solubility behavior of IBF due to the presence of the hydrophilic hydroxyl functional groups largely dispersed within the micronized IBF particles [8,11,41]. To compare and determine the dissolution rate of wet milled micronized IBF particles with dry milled IBF microparticles, the same concentrated excipients were used to develop DPI formulations (Table 1). For instance, formulations FD, FJ and FK were prepared by dry milled IBF microparticles and showed drug dissolution values of 91.4, 96.6 and 93.8%, respectively.

Formulations FD, FJ and FK demonstrated slightly slower IBF dissolution, because the dry milled micronized particles were larger than the wet milled micronized particles and had less surface area to interact with the solvent. Hence, the addition of both excipients helped increase the IBF dissolution in formulation FJ (Figure 7). The dissolution of IBF from all DPI formulations containing leucine (4–6.25%) showed 12.2–18.6% drug release in 2.5 min (Figure 7: FC-b, FF-a, and FF-b). As a result, DPI formulations developed by wet milled and freeze-dried IBF microparticles with leucine presented the fastest drug release (100%) after 120 min compared to the original and milled samples (Figure 7). Leucine is an amphiphilic compound, which contains an α-amino group, an α-carboxylic acid group, and a side chain isobutyl group, making it a less polar aliphatic amino acid in this solvent system. IBF is a weakly acidic drug and the presence of the carboxylic acid group from IBF bonded with the hydrophilic hydroxyl group of leucine enhanced the dissolution in alkaline media (PBS at pH 7.4), compared to acidic media, by forming co-amorphous dispersion [8]. Therefore, this faster drug dissolution of the prepared IBF formulations could be a promising approach for the improvement of IBF bioavailability from inhalable formulations.

## 4. Conclusions

This study reported the successful development of IBF microparticles by wet milling for the preparation of DPI formulations in the presence of flowability enhancers leucine and MgSt. The prepared DPI formulations with leucine and MgSt significantly improved the aerosolization performance, solubility, and dissolution. The presence of large carrier lactose either with the drug particle alone or with the presence of both excipients in a powder mixture provided better aerosolization performance with an increase in FPF from 68 to 37.1. Depending on the concentration, the dispersibility enhancer leucine produced higher FPFs of all formulations compared to those of other formulations containing either MgSt or the combination of MgSt and leucine. The amphiphilic nature of leucine might play a key role in enhancing the FPF of all formulations. The presence of 4–6.25% leucine showed significantly higher IBF dissolution values (98.8% to 100.5%) compared to the original drug (56%) in 2 h. This indicated the formation of a new phase between ibuprofen and leucine in which the existence of a great number of hydrophilic hydroxyl groups distributed with the carboxylic acid group of IBF, which formed a co-amorphous phase in specific time at 37 °C. These hydroxyl groups from leucine successfully increased the drug dissolution by increasing the interaction between drug and solvent. The presence of 2.5% MgSt in the selected DPI formulations led to very good solubility properties. The solubility of IBF microparticles containing MgSt was nearly two-fold higher (252.8 ± 0.6 µg/mL) than that of the particles without MgSt (147.4 ± 1.6 µg/mL). The presence of MgSt at a certain concentration probably formed a new phase between MgSt and IBF by producing an ibuprofen–magnesium complex which significantly enhanced the solubility of poorly water soluble IBF. These results suggest that the data obtained from this study could be useful for successful synthesis of inhalable IBF microparticles by the wet milling method for development of an efficient and stable DPI formulation containing IBF or other poorly soluble drugs with improved aerosolization and dissolution.

## Figures and Tables

**Table 1:** Compositions of developed DPI formulations with carrier lactose and homogeneity of mixtures (n = 3, Mean ± SD).

DPI Formulations	Amount in ‘%’				% Coefficient Variation
	API	Leucine	MgSt	Lactose	
Ibuprofen (Original)	2.5	-	-	97.5	-
FA	2.5	-	-	97.5	2.21 ± 0.41
FB	2.5	-	-	97.5	0.38 ± 0.05
FC-a	2.5	2.5	-	95	2.37 ± 0.29
FC-b	2.5	5.0	-	92.5	2.55 ± 0.52
FE	2.5	2.5	-	95	2.20 ± 0.30
FF-a	2.5	6.25	-	91.25	1.05 ± 0.20
FF-b	2.5	4	-	93.5	0.96 ± 0.14
FG	2.5	5	-	92.5	1.74 ± 0.17
FH	2.5	-	2.5	95	1.50 ± 0.28
FI	2.5	2.5	2.5	92.5	1.49 ± 0.14
FD	2.5	2.5	-	95	4.62 ± 0.26
FJ	2.5	2.5	2.5	92.5	1.92 ± 0.14
FK	2.5	-	-	97.5	2.92 ± 0.32

Note: Here, DPI formulations FD, FJ and FK were prepared by dry milled IBF inhalable sized microparticles. The rest of the DPI formulations were prepared by wet milled IBF inhalable sized microparticles.

**Table 2:** Micronized IBF particle size determination by Malvern Mastersizer 3000 before and after freeze-drying (data provided as Mean ± SD, n = 3).

Name of Micronized Sample	Particle Size (µm) before Freeze-Drying			Particle Size (µm) after Freeze-Drying		
	D_50_	D_[4,3]_	Span	D_50_	D_[4,3]_	Span
Ibuprofen original	71.3 ± 6.96	81.6	3.93	-	-	-
IBF wet milled homogenized	4.02 ± 0.28	4.75	1.66	4.59 ± 0.05	4.93	1.99
IBF wet milled homogenized with leucine (4–5%)	2.03 ± 0.03	2.39	1.80	2.56 ± 0.03	3.85	1.61
IBF wet paste milled homogenized	4.20 ± 0.06	4.58	1.22	4.97 ± 0.03	5.30	1.20
IBF wet paste milled homogenized with leucine (2.5–6.25%)	1.71 ± 0.12	2.15	1.83	1.75 ± 0.14	3.65	0.85

**Table 3:** Powder flow properties obtained from developed DPI formulations from flowability parameter measurements [n = 3, Mean ± SD].

Formulation Code	Bulk Density (g/mL)	Tapped Density (g/mL)	Carr’s Index (%)	Hausner Ratio	Angle of Repose, θ	Flowability
FA	0.147 ± 0.02	0.172 ± 0.01	14.5 ± 0.8	1.17 ± 0.02	34.6 ± 0.1	Good
FB	0.149 ± 0.01	0.173 ± 0.01	13.9 ± 0.6	1.16 ± 0.01	34.2 ± 0.1	Good
FC-a	0.159 ± 0.02	0.181 ± 0.01	12.2 ± 0.2	1.14 ± 0.01	32.6 ± 0.1	Good
FC-b	0.162 ± 0.03	0.181 ± 0.02	10.5 ± 0.3	1.11 ± 0.02	28.7 ± 0.1	Excellent
FE	0.156 ± 0.04	0.179 ± 0.01	12.9 ± 0.4	1.15 ± 0.02	34.3 ± 0.1	Good
FF-a	0.186 ± 0.05	0.205 ± 0.03	9.3 ± 0.7	1.10 ± 0.03	30.4 ± 0.1	Excellent
FF-b	0.191 ± 0.04	0.211 ± 0.04	9.5 ± 0.8	1.10 ± 0.02	27.2 ± 0.1	Excellent
FG	0.178 ± 0.02	0.198 ± 0.02	10.1 ± 0.9	1.11 ± 0.01	28.7 ± 0.1	Excellent
FH	0.185 ± 0.03	0.206 ± 0.02	10.2 ± 0.6	1.11 ± 0.01	27.2 ± 0.2	Excellent
FI	0.153 ± 0.02	0.179 ± 0.01	14.5 ± 0.5	1.17 ± 0.02	34.5 ± 0.3	Good
FD	0.151 ± 0.02	0.175 ± 0.01	13.7 ± 0.5	1.16 ± 0.02	32.9 ± 0.1	Good
FJ	0.161 ± 0.04	0.189 ± 0.02	14.8 ± 0.7	1.17 ± 0.02	32.7 ± 0.4	Good
FK	0.195 ± 0.05	0.241 ± 0.05	19.1 ± 1.0	1.24 ± 0.03	39.9 ± 0.5	Fair

**Table 4:** IBF deposition from DPI formulations using TSI through a Breezhaler^®^ (Mean ± SD, n = 5).

Formulations	Particle Size (µm)	RD (%)	ED (%)	FPF (%)	FPD (µg)
Ibu Original	71.3	96.6 ± 0.8	16.9 ± 1.5	3.7 ± 0.9	4.7 ± 1.1
Wet milled formulations					
FA	4.75	94.8 ± 4.7	80.7 ± 3.5	15.9 ± 1.4	99.4± 6.8
FB	4.20	96.1 ±1.7	83.2 ± 3.4	16.2 ± 2.2	100.4 ± 5.4
FC-a	3.94	94.3 ± 2.8	80.1 ± 3.0	33.2 ± 4.7	233.3 ± 8.3
FC-b	2.56	96.5 ± 2.6	87.5 ± 2.0	27.8 ± 2.0	144.1 ± 5.4
FE	4.75	82.6 ± 2.6	81.8 ± 3.6	6.1 ± 1.9	25.8 ± 8.2
FF-a	3.65	97.5 ± 0.9	90.3 ± 0.6	37.1 ± 3.8	262.4 ± 7.4
FF-b	4.58	99.6 ± 0.7	89.3 ± 1.3	28.8 ± 1.3	266.9 ± 9.5
FG	3.85	89.6 ± 4.5	79.2 ± 6.5	28.8 ± 3.8	139.8 ± 7.9
FH	4.97	87.1 ± 7.5	79.4 ± 4.1	19.0 ± 1.0	90.7 ± 6.3
FI	4.26	92.5 ± 2.8	83.0 ± 1.8	24.8 ± 0.9	144.8 ± 5.9
Dry milled formulations					
FD	4.40	93.9 ± 3.7	80.3 ± 0.6	6.8 ± 2.5	33.9 ± 7.9
FJ		92.2 ± 4.9	80.8 ± 6.0	17.1 ± 1.8	93.4 ± 9.3
FK		89.8 ± 1.9	79.8 ± 4.2	10.6 ± 0.7	52.1 ± 3.4

**Table 5:** Saturated solubility of original, micronized IBF with DPI formulation mixtures in PBS at 22–25 °C (Mean ± SD, n = 3).

Name of the Formulations	Amount in %				Saturated Solubility of Ibuprofen (µg/mL)
	API	Leucine	MgSt	Carrier	
Original and Micronized IBF					
IBF Original					147.4 ± 1.6
IBF Wet milled					159.1 ± 1.1
IBF Dry milled					149.0 ± 0.1
DPI formulation with Wet milled IBF					
FA	2.5	-	-	97.5	157.8 ± 1.1
FB	2.5	-	-	97.5	157.6 ± 0.3
FC-a	2.5	2.5	-	95	162.8 ± 0.1
FC-b	2.5	5.0	-	92.5	167.5 ± 0.1
FF-a	2.5	6.25	-	91.25	167.5 ± 0.7
FF-b	2.5	4	-	93.5	164.1 ± 0.3
FG	2.5	5	-	92.5	162.9 ± 0.1
FH	2.5	-	2.5	95	247.8 ± 1.1
FI	2.5	2.5	2.5	92.5	247.2 ± 0.4
DPI formulation with Dry milled IBF					
FD	2.5	2.5	-	95	146.5 ± 0.1
FJ FK	2.5 2.5	2.5 -	2.5 -	92.5 97.5	252.8 ±0.6 149.4 ±2.5

## Data Availability

Not applicable.

## Abbreviations

IBF—Ibuprofen, L-leucine—leucine, MgSt—Magnesium stearate, PDDS—Pulmonary Drug Delivery System, ATR-FTIR—Attenuated Total Reflectance-Fourier Transform Infrared, DSC—Differential Scanning Calorimetry, TGA—Thermogravimetric Analysis, SEM—Scanning Electron Microscopy, XPRD—X-ray Powder Diffraction, USP—United States Pharmacopoeia, HPLC—High Performance Liquid Chromatography, %—Percent, µg—Microgram, µm—Micrometre, °C—Degree Celsius.
